# Bridging the Gap Between Legal Designation and Management Outcomes: Evidence from a Newly Established Marine Protected Area in Thailand

**DOI:** 10.1007/s00267-026-02622-x

**Published:** 2026-09-24

**Authors:** Suvaluck Satumanatpan, Suhatai Praisankul, Paweekorn Chankum, Pilartrut Singin, Tuanjai Pantorn

**Affiliations:** 1 Faculty of Environment and Resource Studies, Mahidol University, Nakorn Pathom, Thailand; 2 Department of Marine and Coastal Resources, Ministry of Natural Resources and Environment, Bangkok, Thailand; 3 Protected Area Management Center, Moo Koh Racha, Phuket, Thailand

## Abstract

Marine protected areas (MPAs) conserve marine biodiversity, but their effectiveness depends on implementation. This study assessed the Racha Islands MPA, established in 2023 in a tourism-dependent coral-reef setting in Phuket, Thailand. Using the International Union for Conservation of Nature–World Commission on Protected Areas (IUCN-WCPA) management effectiveness framework, the study compiled evidence from May through October 2025, drawing on document analysis, available ecological monitoring information, stakeholder surveys and interviews and field observations and site verification. The 69 respondents comprised 50 local resource users and tourism-related stakeholders and 19 institutional respondents. Performance was evaluated across context, planning, inputs, process, outputs and outcomes. Overall effectiveness was 54.3%, indicating moderate performance. Context (73.3%) and outputs (66.7%) scored highest, reflecting the legal foundation and delivery of many planned activities. Inputs scored lowest (33.3%) owing to financial, human and technical limitations. Outcomes remained moderate (55.6%): most surveyed users perceived threats as broadly unchanged, ecological trends varied across sites and evidence of socio-economic change was limited. The MPA has progressed beyond legal designation, but institutional capacity remains limited and evidence of longer-term effects has not kept pace with management activities. Determining whether observed changes resulted from MPA management was constrained by the short period since establishment, the absence of a pre-designation socio-economic baseline, limited performance indicators and insufficient long-term monitoring. Priorities include predictable financing, adequate staffing and equipment, participatory development of a site-specific management plan and feasible outcome-oriented monitoring. This study provides an early-stage benchmark and lessons for MPA governance in tourism-dependent coastal regions.

## Introduction

Marine ecosystems are increasingly exposed to interacting pressures from climate change, overfishing, coastal development, pollution and expanding tourism. Coral reefs are particularly vulnerable because climate-related disturbances can compound chronic local pressures associated with intensive human use (Murray et al., 2025). In response, marine protected areas (MPAs) have become one of the most widely adopted instruments for conserving marine biodiversity and supporting sustainable resource management (Edgar et al., 2014; Grorud-Colvert et al., 2021).

Despite the rapid expansion of protected-area coverage, MPA effectiveness remains highly variable. Legal designation alone does not ensure effective protection when staffing, funding, enforcement, monitoring and other forms of institutional capacity remain inadequate (Gill et al., 2017; Bailey et al., 2022; Valdivieso et al., 2023; Schéré, 2024). Recent evidence further indicates that an MPA’s stage of establishment and level of protection are important predictors of ecological outcomes, with actively managed areas generally performing more strongly than those that have reached only the initial implementation stage (Horta e Costa et al. (2025)). This distinction underlies continuing concern about ‘paper parks’, in which formal protection is not matched by effective protection and management on the ground (Relano and Pauly, 2023).

Management-effectiveness assessments are therefore important for identifying strengths, weaknesses and implementation gaps across the management cycle. However, management performance must be distinguished from the ecological and social outcomes that protected areas are intended to achieve (Ghoddousi et al., 2022). A recent systematic review also found substantial variation in the scope, objectives and methods used to evaluate MPA management effectiveness, reinforcing the need for transparent and context-sensitive assessments that address multiple dimensions of management (Zafimahatradraibe et al., 2026).

These challenges are particularly pronounced in tourism-dependent coral-reef systems. Marine tourism can generate employment and income and can support conservation initiatives, but excessive visitation, physical contact with corals, fish feeding, anchoring, vessel activity and other tourist behaviours can add pressure to sensitive reef environments (Dong, 2025). Moreover, the effects of multiple activities may interact and accumulate, yet cumulative effects remain incompletely incorporated into many MPA management plans and decision-making processes (Murray et al., 2025). Effective management in these settings therefore requires not only ecological protection measures but also adequate institutional capacity, visitor communication, enforcement, stakeholder participation and coordination among government agencies, tourism businesses, local communities and other resource users. Tourism operators may also serve as management partners by contributing to monitoring, site stewardship and practical conservation activities (Howlett et al., 2022).

Thailand provides an important setting in which to examine these issues because its extensive coral-reef ecosystems support substantial marine-tourism activity. The Racha Islands, located south of Phuket, are intensively used for diving, snorkelling, boating and other recreational activities. The Racha Islands MPA was formally established in 2023 to protect marine and coastal resources within this tourism-dependent setting. Its establishment provided a legal foundation for conservation but also created the challenge of translating general protection objectives into site-specific, adequately resourced and participatory management.

Because the assessment was conducted approximately two years after formal establishment, the Racha Islands MPA provides an opportunity to examine management during an early stage of implementation. An early assessment can establish an initial performance benchmark, identify institutional and governance constraints and distinguish management activities and outputs from ecological and socio-economic outcomes that may require longer periods to become detectable. This distinction is particularly important where baseline information and long-term monitoring systems remain underdeveloped.

This study evaluates the management effectiveness of the Racha Islands MPA using the IUCN-WCPA management-effectiveness framework. Based on evidence compiled from May through October 2025 through document analysis, stakeholder surveys and interviews conducted from May through July, field observations and follow-up site verification and available ecological monitoring information, the assessment examines six components of the management cycle: context, planning, inputs, process, outputs and outcomes. Specifically, the study evaluates performance across these components, identifies the principal institutional and governance constraints on implementation and examines the extent to which management outputs are accompanied by evidence of ecological and socio-economic outcomes. In doing so, it provides an early-stage benchmark for the Racha Islands MPA and contributes empirical evidence on the management of newly established MPAs in tourism-dependent settings in Southeast Asia.

## Study Area and Management Context

### Study Area

**Fig. 1:**
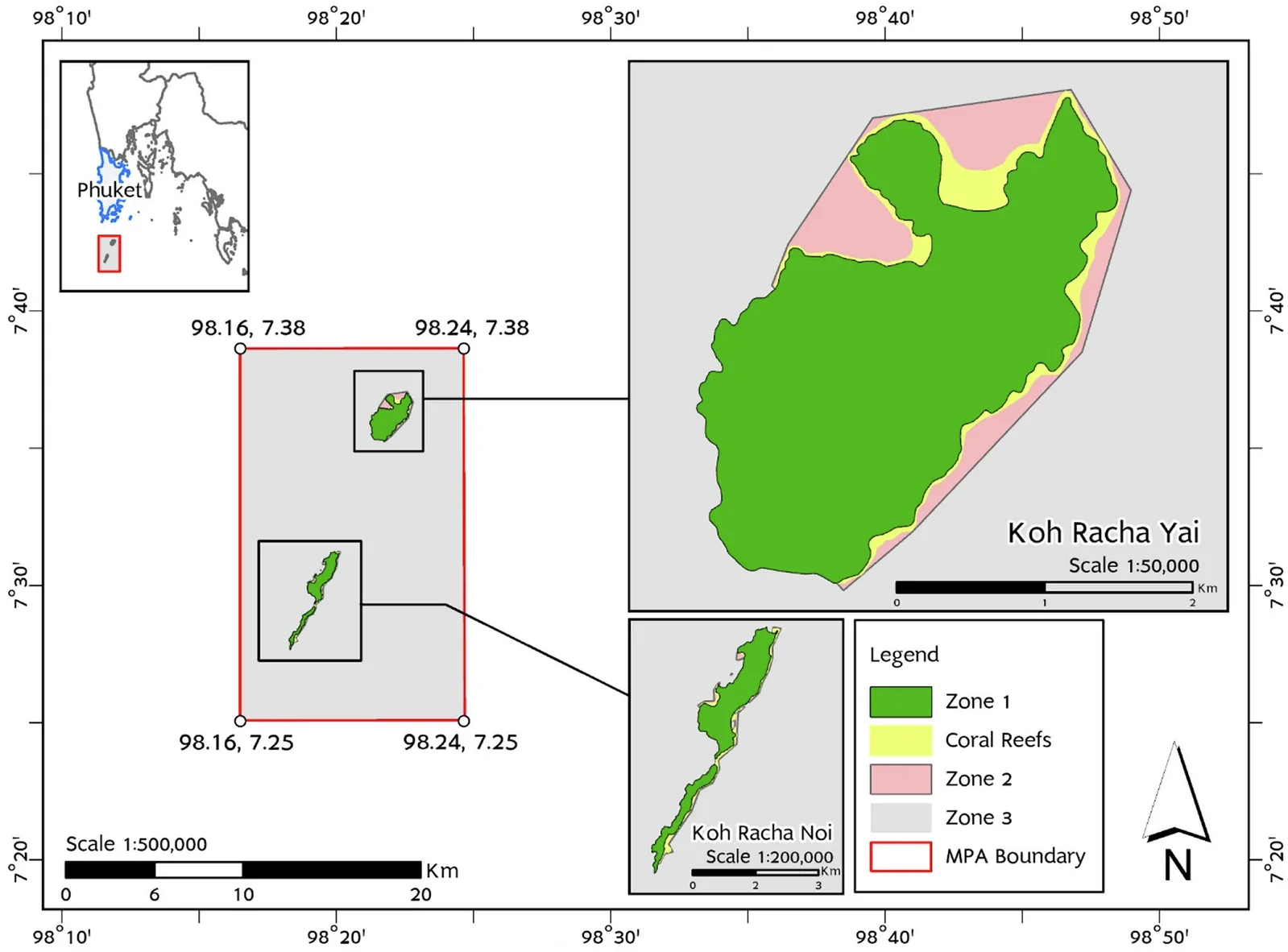
Location and management zoning of the Racha Islands Marine Protected Area in the Andaman Sea, Phuket Province, Thailand. The map shows Koh Racha Yai and Koh Racha Noi, the MPA boundary, coral-reef habitats and management Zones 1–3; the locator inset shows the islands’ position south of Phuket. The MPA boundary and management zones are based on the Ministry of Natural Resources and Environment (2023). Map prepared by Ms. Somruedee Kawlomlerd, Faculty of Environment and Resource Studies, Mahidol University

The study was conducted in the Racha Islands Marine Protected Area (MPA), comprising Racha Yai and Racha Noi, located approximately 21–25 km south of Phuket in the Andaman Sea, Thailand (Fig. 1). The MPA covers 375.359 km² (37,535.9 ha), based on the legally defined outer boundary (Ministry of Natural Resources and Environment 2023). Racha Yai is the larger island and supports a small resident community and tourism facilities, including accommodation and restaurants. Racha Noi is uninhabited and is used primarily for nature-based tourism and marine recreation. Both islands support coral reef ecosystems and are popular destinations for diving and other marine tourism activities in Phuket.

Tourism activities are intensive, particularly scuba diving, snorkelling, recreational boating and other water-based activities. Many visitors participate in day trips, resulting in substantial vessel activity around beaches, reef areas and dive sites. Reported tourism-related pressures include anchor damage to coral reefs, physical contact with corals by divers and snorkellers, fish feeding, collection of corals and other marine organisms, waste and wastewater discharge and boat movements across shallow reef areas. Together with coral bleaching, these pressures have increased concerns regarding coral reef condition and tourism management (DMCR, 2021).

### Management Context

The Racha Islands MPA was formally established in 2023 under Section 22 of Thailand’s Act on the Promotion of Marine and Coastal Resources Management, B.E. 2558 (2015). The ministerial notification was issued on 17 February 2023 and published in the Royal Thai Government Gazette on 13 March 2023. Under Clause 14, the notification remains in force for five years from the date on which it took effect (Ministry of Natural Resources and Environment, 2023).

The protected area is divided into three management zones. Zone 1 covers the terrestrial areas of Racha Yai and Racha Noi from the shoreline inland. Zone 2 extends from the shoreline to the natural coral reefs and adjacent marine areas. Zone 3 encompasses the offshore waters surrounding both islands within the legally defined outer boundary. Management measures vary among the three zones and include restrictions on waste and wastewater discharge, coastal construction and sediment-generating activities, anchoring on coral reefs, fisheries, fish feeding and the collection or possession of marine organisms for tourist display. Tourism activities that may damage coral reefs are also restricted. Vessel entry, mooring and tourism operations are subject to specified conditions and the Director-General of the Department of Marine and Coastal Resources may limit visitor and vessel numbers according to the ecological carrying capacity of the area.

Operational responsibility rests primarily with the Racha Islands Protected Area Management Centre under the Department of Marine and Coastal Resources (DMCR). The Centre is responsible for operational planning, ecological monitoring, enforcement, conservation and restoration, public communication and stakeholder engagement. Its work is supported by other government agencies, local authorities, tourism operators, community groups and volunteer divers. At the time of the assessment, the Centre had six staff members and limited operational assets, including two patrol vessels, one of which required repair, a vehicle and a temporary field office on Racha Yai (DMCR, 2025).

The general five-year management plan (2024–2028) (DMCR, 2024) covers administration, ecological monitoring and evaluation, enforcement and patrol, coral reef conservation and restoration, mooring-buoy installation and maintenance, sustainable tourism and stakeholder participation. At the time of data collection, implementation remained at an early stage. The present assessment therefore examines how the legal designation and management plan were being translated into institutional arrangements, operational capacity, management processes and early management outputs and outcomes.

## Materials and Methods

### Analytical Framework

This study applied the International Union for Conservation of Nature–World Commission on Protected Areas (IUCN-WCPA) management effectiveness framework to evaluate the management effectiveness of the Racha Islands Marine Protected Area (Hockings et al., 2006). The IUCN-WCPA framework remains one of the most widely applied approaches for assessing the management effectiveness of protected areas and marine protected areas worldwide (Zafimahatradraibe et al., 2026). Assessment indicators were adapted from the Management Effectiveness Tracking Tool (METT) (WWF, 2007) and the marine management scorecard developed by Staub and Hatziolos (2004).

The final assessment framework comprised 43 indicators distributed across the six components of the IUCN-WCPA management cycle: context, planning, inputs, process, outputs and outcomes. Table 1 summarises the number of indicators and principal assessment focus within each component. Together, the indicators provide a systematic basis for evaluating performance across the full protected-area management cycle. The complete indicator set and indicator-specific scoring criteria are provided in Table A1.

**Table. 1:** Distribution and principal assessment focus of the management-effectiveness indicators

IUCN-WCPA component	No. of indicators	Examples of assessment focus
Context	5	Legal status, boundaries, resource values, threats and stakeholders
Planning	8	Management policy, objectives, planning, coordination and participation
Inputs	7	Budget, personnel, equipment, facilities, training and information
Process	11	Governance, coordination, participation, enforcement and monitoring
Outputs	6	Implementation of management activities and delivery of planned actions
Outcomes	6	Environmental and socio-economic outcomes

### Data Collection

The study employed a mixed-methods approach that integrated document analysis, stakeholder questionnaire surveys, semi-structured interviews, in-depth interviews, field observations and available ecological monitoring information.

First, document analysis was conducted using policy documents, legal instruments, management plans and official reports related to the establishment and governance of the Racha Islands MPA. These materials provided evidence on the legal and institutional framework, management objectives, planning arrangements, implementation activities and available management resources.

Second, stakeholder surveys and interviews were conducted from May through July 2025 with 69 respondents selected through purposive sampling (Table 2). Stakeholder groups were first identified through a review of the ministerial notification establishing the Racha Islands MPA, particularly its management measures, responsible authorities and affected resource-use groups. This review was supplemented by preliminary consultations with DMCR personnel familiar with the area. Within these groups, individuals were eligible if they had continuing, direct roles in the use, management, or monitoring of the MPA. Local resource users and tourism-related respondents were selected from people engaged in small-scale fishing, community activities, or tourism-related operations in the Racha Islands area. Institutional respondents were selected because their official or professional responsibilities involved MPA establishment, administration, enforcement, ecological monitoring, inter-agency coordination, or technical advice. The final sample comprised 50 local resource users and tourism-related stakeholders and 19 institutional respondents.

**Table. 2:** The composition of stakeholder respondents included in the study

Respondent category	Stakeholder group	Number of respondents
Local resource users and tourism-related stakeholders (*n* = 50)	Small-scale fishers	21
	Community members	3
	Tourism operators	26
Institutional respondents (*n* = 19)	DMCR officials	14
	Officials from other government agencies	3
	Academic experts	2
Total		69

Questionnaire surveys, supplemented by semi-structured interviews, were conducted with the 50 local resource users and tourism-related stakeholders. This combination was chosen to obtain comparable responses across a relatively large and heterogeneous group while allowing respondents to explain their experiences. The questionnaires examined awareness of the MPA and its ecological importance, perceptions of threats and environmental change, participation in planning and management, understanding of regulations, compliance, livelihood effects and satisfaction with management. The semi-structured questions provided additional explanations of respondents’ experiences and perceptions of management implementation.

Semi-structured in-depth interviews were conducted with 19 institutional respondents using a predefined interview guide. This method was used because respondents’ role-specific knowledge required flexible and detailed exploration of governance arrangements, inter-agency coordination, resource allocation, management implementation, operational constraints, stakeholder participation and opportunities for management improvement beyond the standardised stakeholder questionnaire.

Appendix A contains the assessment framework and scoring documentation: Table A1 presents the complete indicator set and indicator-specific scoring criteria and Table A2 presents the final indicator scores, evidence sources and scoring rationales. Appendix B summarises the questionnaire and semi-structured interview guide used with local resource users and tourism-related stakeholders, while Appendix C summarises the semi-structured in-depth interview guide used with institutional respondents.

Ethical approval for this study was granted by the Central Institutional Review Board of Mahidol University.

Third, field observations were conducted from May through October 2025. They began during stakeholder data collection and continued through follow-up site visits after the formal surveys and interviews ended in July. These visits were used to verify documentary and interview evidence, observe tourism activities, management measures, enforcement practices, management facilities and environmental conditions and clarify specific issues with relevant stakeholders. The follow-up discussions were not treated as additional formal interviews and did not alter the reported sample of 69 respondents. Available ecological monitoring information from DMCR and external ecological studies was also used, where relevant, to support the interpretation of resource conditions and management outcomes.

### Management Effectiveness Assessment

Management effectiveness was assessed using a structured scoring approach based on the IUCN-WCPA framework. Each of the 43 indicators was evaluated using the four-point scale shown in Table 3. Indicator-specific scoring criteria are provided in Table A1.

**Table. 3:** Indicator scoring scale and percentage-based performance classification

Measure	Score or range	Interpretation
Indicator score	0	No relevant management action or supporting evidence
Indicator score	1	Limited or initial action
Indicator score	2	Partial implementation
Indicator score	3	Effective implementation supported by clear evidence
Performance	<20%	Very low
Performance	20– < 40%	Low
Performance	40– < 60%	Moderate
Performance	60– < 80%	Good
Performance	≥80%	Excellent

Evidence relevant to each indicator was compiled and cross-checked across the available data sources. Documentary evidence was used primarily to assess legal, institutional and planning arrangements; questionnaire responses supported the assessment of stakeholder awareness, participation, compliance, livelihood effects, satisfaction and perceived environmental change; and interviews provided evidence on governance, coordination, implementation and operational constraints. Field observations and available ecological information were used to verify management activities and environmental conditions.

Scores were not derived directly from any single source. Where evidence differed among sources, the research team jointly reviewed the relevant information and reached a consensus on the final score, taking into account the relevance, consistency and credibility of the available evidence. Table A2 presents the final indicator scores, evidence sources and the basis for each score.

A percentage score was calculated separately for each management component using the following equation:

#### Component Score (%) = [Sum of Indicator Scores Within the Component ÷ Maximum Possible Score for that Component] × 100

The maximum possible score for each component was calculated as the number of indicators within that component multiplied by three. This procedure allowed comparison among components despite differences in the number of indicators.

The overall management effectiveness index was calculated by dividing the sum of the scores for all 43 indicators by the maximum possible total score of 129 and multiplying the result by 100:

#### Overall Management Effectiveness Index (%) = [Sum of All Indicator Scores ÷ 129] × 100

Percentage scores were interpreted using the five performance categories shown in Table 3. This classification was developed for the present study to facilitate interpretation and was informed by approaches used in global assessments of protected-area management effectiveness (Leverington et al., 2010a).

Post-assessment governance and planning developments described in the Discussion were documented from the relevant legal and administrative records. Because these developments occurred after the assessment period from May through October 2025, they were not included in the management-effectiveness scoring.

### Limitations

Several limitations should be considered when interpreting the findings. First, the Racha Islands MPA had been formally established approximately two years before data collection. The study therefore evaluated management during an early stage of implementation, when institutional arrangements, enforcement practices, monitoring systems and stakeholder participation were still developing. The findings should consequently be interpreted as an assessment of initial management performance rather than evidence of long-term ecological or socio-economic outcomes.

Second, respondents were purposively selected using the predefined criteria described in Section 3.2. This approach provided access to stakeholders with direct and recurrent experience of MPA use, management, or monitoring but was not intended to produce a statistically representative sample of all stakeholder groups. In addition, some indicators—particularly those concerning awareness, compliance, satisfaction and perceived environmental change—relied partly on respondents’ perceptions. Triangulation with documentary evidence, field observations and available ecological information reduced, but did not eliminate, the potential for subjectivity.

Third, tourists were not included because the sampling design focused on respondents with continuing, direct involvement in the use, management, or monitoring of the MPA and who could comment on management implementation over time. Most visitors to the Racha Islands are transient or short-stay tourists; adequately representing this group would have required a separate multilingual sampling design covering different visitor types and seasons, which was beyond the scope of the present early-stage assessment. Consequently, the study could not directly assess visitor awareness of the protected-area designation, the effectiveness of signage and operator briefings, or tourists’ understanding of MPA regulations. Future assessments should incorporate multilingual visitor surveys and repeated ecological and socio-economic monitoring to evaluate changes as management implementation matures.

## Results

### Overall Management Effectiveness of the Racha Islands Marine Protected Area

**Fig. 2:**
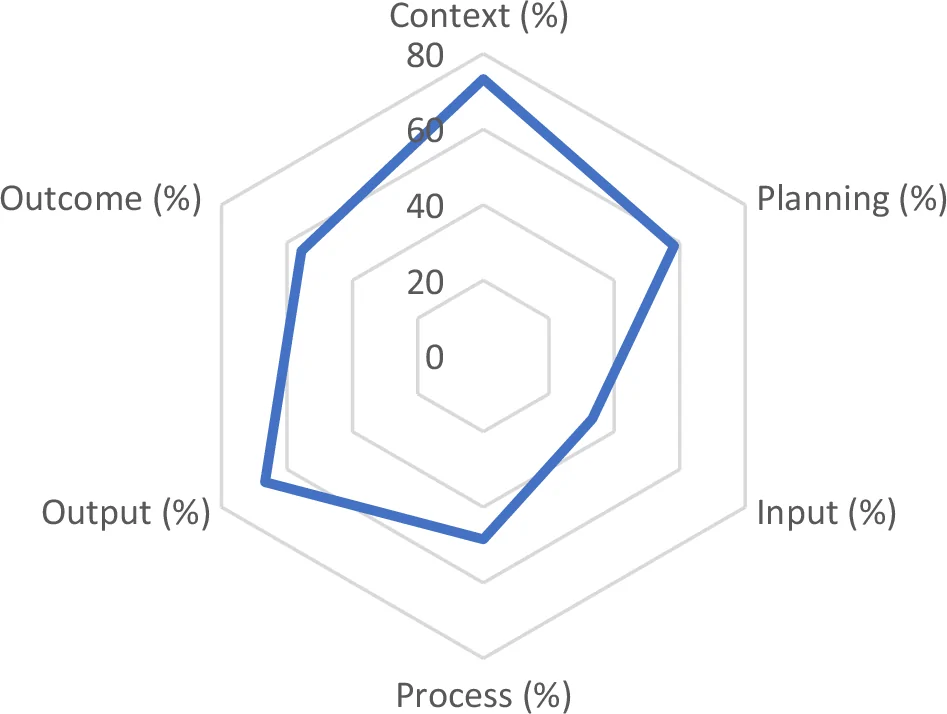
Relative performance of the six management effectiveness components of the Racha Islands MPA

The overall management effectiveness score of the Racha Islands MPA was 54.3%, indicating moderate performance. Context (73.3%) and outputs (66.7%) were rated good, planning (58.3%), process (48.5%) and outcomes (55.6%) were moderate and inputs (33.3%) were low. This pattern shows that the legal and planning foundations and the delivery of management activities were stronger than the resources available, the operation of management processes and the evidence of achieved outcomes. Table 4 summarises the component scores, strengths and constraints, while Fig. 2 illustrates the relative performance of the six management components.

**Table. 4:** Summary of management effectiveness assessment results for the Racha Islands MPA

Component and score	Performance level	Key strengths	Key limitations or challenges
Context: 11/15 (73.3%)	Good	Legal designation; high awareness of ecological and use values and continuing threats	Operational boundaries and zones are unclear to many users; physical markers are incomplete; threats have not clearly declined
Planning: 14/24 (58.3%)	Moderate	General five-year management plan; ecologically informed design and zoning; alignment with relevant agencies	Few quantitative targets; annualised operational planning; stakeholders had no substantive role in preparing the management plan
Inputs: 7/21 (33.3%)	Low	Ecological baseline information; some support from private-sector and local stakeholders	Insufficient and unstable funding; too few staff; limited skills, equipment and facilities; no socio-economic baseline
Process: 16/33 (48.5%)	Moderate	Regular implementation reporting; some use of research and monitoring; participation in selected activities	Limited external communication; reactive training and maintenance; procedural enforcement constraints; informal conflict resolution
Outputs: 12/18 (66.7%)	Good	More than 75% of planned activity categories implemented; most outputs delivered broadly on time	Some outputs incomplete or externally dependent; uneven stakeholder understanding; recurring violations; limited institutional understanding of the plan
Outcomes: 10/18 (55.6%)	Moderate	Stable or improving ecological conditions at several sites; satisfaction above 80%; emerging collaboration	Threats broadly unchanged; ecological trends inconsistent; little socio-economic change; limited indicators and baselines; collaboration not institutionalised
Overall: 70/129 (54.3%)	Moderate	Strong legal foundation and delivery of many planned activities	Resources, operational processes and evidence of ecological and socio-economic outcomes lagged behind legal designation and activity delivery

### Strong Legal Foundation and Stakeholder Awareness

The context component achieved the highest score (73.3%), indicating a good foundation for management. The MPA had been legally designated and surveyed local resource users and tourism-related stakeholders showed substantial awareness of its ecological and use values: 60% identified its habitat function, 48% identified its importance for marine tourism and more than 90% recognised continuing threats to marine and coastal resources.

Relevant government, tourism, fishing, community, conservation and academic groups had been identified and were involved in some consultations or management activities. Management responses to recognised threats included patrols, mooring buoys, zoning and other measures. However, stakeholder engagement was not regular or comprehensive and the available evidence did not show that threats had clearly declined.

Operational boundary clarity remained a major weakness. Although the legal boundary was mapped, physical markers were absent or incomplete and 54.8% of surveyed users considered the boundary unclear, particularly around the coral-reef zone. Thus, the strong legal foundation had not yet translated into equally clear on-site communication of the protected area and its zones.

### Planning Frameworks are Established, but Participation Remains Limited

The planning component achieved a moderate score of 58.3%. The Act on the Promotion of Marine and Coastal Resources Management, B.E. 2558 (2015), together with the general five-year management plan (2024–2028), provided clear strategic direction covering administration, monitoring, protection, restoration and stakeholder participation. The protected-area design and three management zones reflected coral reefs, coastal habitats, ecological connectivity and differentiated use controls. Management responsibilities were also aligned with relevant fisheries and marine transport authorities, with no major duplication or conflict identified.

Important planning gaps nevertheless remained. Management objectives were stated but generally lacked quantitative targets and measurable outcome indicators. Annual plans specified activities and timing, but operational details were incomplete and reliance on annual planning limited multi-year continuity. Plans addressed threat reduction, restoration and participation, but implementation was incomplete because of budget and staffing constraints.

Stakeholder participation in management planning was particularly weak. Although the general five-year management plan included stakeholder participation as a strategic area, stakeholders had been consulted only during designation and had no substantive role in preparing either the general five-year management plan or the annual implementation plan for fiscal year 2025 (Racha Islands Protected Area Management Center, 2025). Interview respondents were generally unaware of the annual implementation plan. PL8 (Table A2) therefore received a score of 0, distinguishing the inclusion of participation in policy documents from actual stakeholder involvement in planning and decision-making.

### Resource Limitations Constrain Management Implementation

Resource limitations were the most significant constraint on management effectiveness. Inputs received the lowest component score (33.3%), while process performance was moderate (48.5%). The contrast indicates that implementation was constrained even though formal management structures and plans were in place.

The management budget was insufficient for routine administration and field operations and depended on annual government allocations, with no secure multi-year financing. Six staff members were responsible for island-based fieldwork, vessel operations, patrols, technical tasks and administration. Essential equipment and facilities were also inadequate: of the Centre’s two patrol vessels, one required repair, while the other—the only operational vessel—experienced frequent breakdowns. The temporary island office was not fully operational. Ecological baseline information and external support were available, but socio-economic baseline data were lacking and stakeholder support remained uneven.

Some management processes performed comparatively well. Implementation progress was monitored and reported at approximately 4- to 6-month intervals. Management staff also reported referring to findings from external ecological studies during implementation. Although stakeholders had no substantive role in preparing the annual implementation plan, 58% of surveyed users reported participating in management activities. Participation in these activities nevertheless remained uneven across stakeholder groups. Staff training mainly addressed immediate needs, maintenance was reactive and uncertainty about enforcement procedures reduced effective implementation. The advisory committee appointed in March 2025 had not yet begun operating; conflict resolution remained largely informal and about 63% of surveyed users considered public communication ineffective or insufficient.

### Outputs Achieved Despite Resource Limitations

The outputs component achieved a good score of 66.7% despite resource limitations. More than 75% of planned activity categories had been implemented and activities were generally completed within the planned timeframe. Most intended outputs and services were delivered, although some remained incomplete or depended on support from partner agencies and stakeholders.

Implemented activities included mooring-buoy work, surveillance, underwater clean-ups, coral restoration and stakeholder and public-awareness activities. These outputs show that management plans had begun to be translated into operational action during the early stage of MPA implementation.

Knowledge and compliance results were more mixed. Approximately 54.3% of surveyed users reported a substantial or strong understanding of MPA management. Self-reported compliance was high (83.7%), but recurring violations, including anchoring on coral reefs and fish feeding, were still reported. Fewer than half of institutional respondents demonstrated an understanding of the management plan, indicating an important internal knowledge gap.

### Outcomes Remain Moderate and Uneven

The outcomes component achieved a moderate score of 55.6%, but evidence of realised change was uneven. Some progress toward management objectives was evident, yet the lack of quantitative targets prevented broad verification. Most surveyed users perceived the overall level of threats as broadly unchanged. Ecological monitoring indicated stable or improving conditions at several sites, but deterioration at some coral areas meant that an MPA-wide trend could not yet be established. Socio-economic outcomes were also limited: most respondents reported little change in income or related conditions and no pre-designation socio-economic baseline was available. In contrast, more than 80% of surveyed users were satisfied with management and enforcement. Stakeholders and agencies collaborated in several activities, but these arrangements remained largely informal and were not supported by a fully operational joint-management mechanism.

The outcome assessment therefore relied partly on stakeholder perceptions and short-term ecological information rather than on a comprehensive set of repeated ecological and socio-economic indicators. The findings represent early indications of performance and do not yet demonstrate long-term conservation or livelihood outcomes attributable to the MPA.

## Discussion

The findings reveal a central challenge in protected-area governance: legal designation and a general, non-site-specific management plan do not necessarily translate into effective site-level implementation or demonstrable outcomes. Although the Racha Islands MPA benefits from a strong legal foundation and has generated a range of management outputs, its moderate overall performance reflects an imbalance between relatively strong context and outputs and weaker management inputs, partially functioning management processes and still-emerging ecological and socio-economic outcomes. This interpretation is consistent with evidence that formal designation and initial implementation are insufficient when staffing, funding, enforcement and monitoring capacity remain limited (Gill et al., 2017; Bailey et al., 2022; Valdivieso et al., 2023; Schéré, 2024), as well as with recent work distinguishing management actions and stages of establishment from demonstrated ecological and social outcomes (Ghoddousi et al., 2022; Relano and Pauly, 2023; Horta e Costa et al.,, (2025)).

### Early-Stage Performance of a Newly Established Marine Protected Area

The assessment captures the Racha Islands MPA at an early stage, approximately two years after its formal establishment. Its uneven performance across the six management components indicates that implementation has not progressed at the same rate throughout the management cycle. Context and outputs achieved the highest scores, whereas inputs received the lowest score; planning, process and outcomes remained moderate. This pattern shows that the MPA has progressed beyond legal designation and initiated a substantial range of management activities, but has not yet developed the resources, operational capacity, or evidence base required to demonstrate longer-term effectiveness. Assessing performance across the full management cycle is therefore important to avoid equating the delivery of management activities and outputs with ecological or socio-economic success (Grorud-Colvert et al., 2021; Ghoddousi et al., 2022).

Comparable patterns have been observed when legal designation and rapid expansion of protected-area coverage outpace improvements in management effectiveness and the development of institutional and operational capacity (Leverington et al., 2010b; Amkieltiela et al., 2022). For the Racha Islands MPA, legal designation and the general five-year management plan (2024–2028), developed for MPAs generally, provided an initial administrative foundation. However, this management plan was not tailored to site-specific ecological conditions, resource-use patterns, or tourism pressures. At the time of the assessment, this foundation remained incomplete: the general five-year management plan had not yet been translated into a site-specific management plan developed with stakeholder participation, operational resources were limited and systems for demonstrating ecological and socio-economic outcomes remained underdeveloped. Recent meta-analytic evidence indicates that MPAs at the actively managed stage are associated with stronger ecological outcomes than those classified only as implemented, particularly when coupled with higher levels of protection (Horta e Costa et al.,, (2025)). This distinction is important for interpreting the Racha Islands assessment: the general five-year management plan and visible management outputs represented genuine progress but did not yet demonstrate conservation impact. Regional experience from the Coral Triangle similarly demonstrates that MPA effectiveness depends on translating conservation objectives into sustained and adequately resourced management action (White et al., 2014). Strengthening institutional capacity and outcome-oriented monitoring will therefore be essential as implementation progresses.

### Institutional Capacity and Implementation Constraints

Institutional capacity emerged as the primary constraint on management effectiveness in the Racha Islands MPA, as reflected in the low input score. These constraints involved not only shortages of financial, human and technical resources but also limited stability and operational continuity. Management depended on annual government allocations, staffing was insufficient for the range of island-based and administrative responsibilities and essential equipment and facilities were inadequate. The lack of systematic capacity-building and preventive-maintenance arrangements further contributed to reactive rather than sustained implementation. Although implementation reporting and the use of external ecological research performed comparatively well, limited capacity constrained consistent enforcement, stakeholder engagement, public communication and the systematic integration of monitoring evidence into management decisions.

This finding is consistent with global and recent evidence identifying institutional capacity as a critical determinant of protected-area performance and showing that staffing, finance, equipment, monitoring and institutional support shape the delivery of management outputs (Gill et al., 2017; Bailey et al., 2022; Valdivieso et al., 2023; Schéré, 2024). Comparable capacity constraints have been documented in protected areas in Taiwan and Thailand (Lu et al., 2012; Satumanatpan et al., 2014). More recent evidence from 36 priority MPAs in Indonesia further demonstrates that staff capacity depends not only on personnel numbers but also on clear roles and competencies aligned with operational needs (Capriati et al., 2026). In the Racha Islands, support from partner agencies, tourism operators and other stakeholders enabled many planned activities to be implemented. However, dependence on such supplementary support also suggests that the continuity of management outputs may remain vulnerable.

The contrast between planning performance (58.3%) and inputs (33.3%) illustrates a mismatch between management ambitions and available implementation capacity. Improving effectiveness, therefore, requires more than further refinement of plans and regulations. Priority should be given to predictable financing, adequate staffing and equipment, systematic and competency-based staff development and preventive-maintenance arrangements. Strengthening these foundations would help shift management from partly reactive implementation toward more sustained, adaptive and outcome-oriented management.

### Tourism Pressures on Coral Reef Governance

Tourism is economically important to the Racha Islands but also creates substantial management demands at sensitive coral-reef sites. Respondents and field observations identified continuing pressures associated with anchoring on coral reefs, fish feeding, physical contact with corals and intensive vessel and recreational activity. The recurrence of these activities despite zoning, mooring buoys, patrols and other management measures suggests that this governance challenge lies not only in establishing regulations but also in communicating spatial restrictions, facilitating compliance and enforcing rules across a heavily used marine environment. Because the study did not quantify visitor intensity or establish causal relationships between tourism activities and ecological change, these findings should be interpreted as evidence of continuing governance pressures rather than direct evidence of tourism-driven reef degradation.

A recent review indicates that physical contact with corals, excessive visitation, fish feeding and other tourist behaviours remain recurring pressures in marine destinations and that effective responses must address behavioural and contextual barriers rather than rely on information provision alone (Dong, 2025). The challenge is compounded when repeated and interacting pressures are not considered cumulatively; a recent global review found that cumulative effects remain incompletely integrated into many MPA management plans and decision-making processes (Murray et al., 2025). Research from Koh Tao, Thailand, has demonstrated associations between intensive diving activity, physical coral damage and coral disease prevalence (Lamb et al., 2014). More recent longitudinal evidence from the same island showed that a temporary tourism halt was insufficient to reverse deteriorating coral health and that reef condition reflected the combined influence of climatic and chronic anthropogenic pressures (Baruffaldi et al., 2026). Taken together, the Koh Tao studies caution against attributing changes in reef condition to tourism intensity alone. More broadly, recent scholarship on protected-area tourism emphasises that balancing ecological protection with the economic benefits of tourism requires multi-stakeholder governance involving conservation agencies, tourism businesses, local communities and other resource users (Sarhan et al., 2024). The experience of the Racha Islands is consistent with this broader governance challenge, although the site-specific ecological effects of tourism within the MPA require further quantitative investigation.

Tourism operators should therefore be viewed not only as regulated resource users but also as potential management partners. Diving and tourism groups in the Racha Islands already contributed to activities such as mooring-buoy work, underwater clean-ups and conservation initiatives, indicating that their involvement can supplement limited government capacity. Evidence from the Great Barrier Reef similarly demonstrates that conservation activities can be integrated into tourism operations to promote site-based reef stewardship (Howlett et al., 2022). More structured arrangements for operator briefings, communication of zones and regulations, mooring-buoy maintenance, reporting of violations and collaborative monitoring could help convert existing participation into a more sustained form of stewardship. However, because short-stay and international tourists were not included in the assessment, the effectiveness of signage, operator briefings and visitor communication remains uncertain and should be examined in future evaluations.

### From Consultative Participation Toward Collaborative Governance

Stakeholder participation in the Racha Islands MPA varied across stages and forms of management. Stakeholders were consulted during the designation process and 58% of surveyed users reported participating in management activities. At the time of the assessment, however, the general five-year management plan had not yet been translated into a site-specific plan through a participatory process and the advisory committee appointed in March 2025 had not yet begun operating. Participation was therefore neither absent nor fully collaborative; rather, it was concentrated primarily in consultation and selected operational activities, with limited formal stakeholder influence over management priorities and decision-making. This distinction is important because participation in implementing management activities does not necessarily constitute participation in planning, agenda-setting, or shared governance (Mast et al., 2025).

High levels of satisfaction with management and self-reported compliance may indicate a degree of acceptance of the MPA but should not be interpreted as evidence of shared decision-making. Stakeholders and agencies cooperated in activities such as mooring-buoy work, clean-ups and conservation initiatives, yet these relationships remained largely informal and were not supported by a fully operational collaborative mechanism. Meaningful engagement can strengthen compliance, local support and legitimacy, but these benefits depend on whether participation provides genuine influence over decisions (Bennett and Dearden 2014; Garcia Rodrigues et al., 2024). In Southeast Asian MPAs, governance effectiveness has similarly been linked to dialogue, trust-building, communication and institutional mechanisms that facilitate interaction among state and non-state actors (Ho et al., 2014).

The advisory committee subsequently held its first formal meeting in Phuket in July 2026, providing an institutional pathway for moving from consultation and activity-based participation toward collaborative governance (Racha Islands Protected Area Management Center, 2026). This post-assessment development represents an important step toward establishing a more structured mechanism for stakeholder engagement, but its contribution to collaborative governance will depend on representative membership, a clear mandate, regular meetings, access to management information and transparent mechanisms through which stakeholder input is formally considered and can influence decisions. Recent experience from a remote coral-reef sanctuary highlights the role of an advisory council in structuring inclusive dialogue, co-producing recommendations and supporting stakeholder outreach (Dunning et al., 2026). A recent global analysis also found that shared-governance arrangements were associated with stronger ecological performance than state-only governance, while emphasising the need for representative and contextually appropriate institutional design (Mast et al., 2025). For the Racha Islands, sustained participation by government agencies, local communities, fishers, tourism operators and conservation groups will be necessary to convert existing but largely informal contributions into collaborative planning, shared responsibility and joint oversight of agreed management activities.

Future management-effectiveness assessments should also adopt a more participatory process in which local stakeholders help interpret monitoring evidence, discuss indicator scores and identify management priorities. The Marine Protected Area Management Effectiveness Assessment Tool (MPA MEAT), developed for use in the Philippines, provides a practical example of using assessment to strengthen local understanding and institutional capacity (MPA Support Network, 2011). Its criteria would require adaptation to Thailand’s legal and institutional context, but its participatory principle is directly relevant to the Racha Islands.

### From Management Outputs to Measurable Conservation Outcomes

One of the most notable findings is the contrast between good performance in management outputs (66.7%) and only moderate performance in outcomes (55.6%). More than 75% of planned activity categories had been implemented and most intended outputs had been delivered, although some remained incomplete or depended on external support. However, most surveyed users perceived threats as broadly unchanged, ecological trends were inconsistent across sites and most respondents reported little change in income or related socio-economic conditions. High stakeholder satisfaction represents an important positive social result but does not, by itself, demonstrate broader ecological or socio-economic effectiveness. This contrast illustrates why MPA effectiveness must be assessed not only through the delivery of management activities but also through evidence of resulting environmental and social change (Grorud-Colvert et al., 2021; Ghoddousi et al., 2022; Valdivieso et al., 2023).

The difference between outputs and outcomes is a recurring challenge in protected-area management. Recent research on coral-reef conservation initiatives shows that enabling conditions and management arrangements do not themselves guarantee positive outcomes; their effects depend on how they are operationalised, sequenced and connected through management action (Salaün et al., 2026). The MPA Guide likewise distinguishes an MPA’s stage of establishment and level of protection from the ecological and social outcomes it ultimately achieves (Grorud-Colvert et al., 2021). This distinction between management action and measurable outcomes also informs wider debates over so-called ‘paper parks’ (Relano and Pauly, 2023). However, the Racha Islands MPA should not be characterised simply as a paper park because legal establishment has been followed by substantive management activities and outputs. It is more accurately understood as an early-stage MPA in which implementation has advanced more rapidly than the capacity to demonstrate its effects. Attribution remains constrained by the short period since designation, the absence of a pre-designation socio-economic baseline, limited performance indicators, mixed ecological trends and partial reliance on stakeholder perceptions. Consequently, the available evidence cannot establish that observed ecological or socio-economic changes were caused by MPA management.

Strengthening outcome-oriented monitoring should therefore focus on a feasible set of measurable and repeatable indicators linked directly to management objectives. Given the MPA’s limited financial and human resources, this system should build on existing DMCR monitoring and, where appropriate, data collected under standardised protocols by partner agencies, researchers, tourism operators and local stakeholders, rather than create an overly complex new programme. Clearly defined ecological, socio-economic and governance indicators, together with baseline values, monitoring intervals, institutional responsibilities and transparent reporting, would provide the feedback required to connect monitoring evidence with adaptive decision-making (Williams and Brown 2014; Murray et al., 2025).

The preparation of a site-specific management plan for the Racha Islands MPA was already required under Clause 13 of the ministerial notification establishing the protected area (Ministry of Natural Resources and Environment 2023). Following completion of this assessment, DMCR began preparing the required plan and used the assessment findings to inform the planning process. The assessment, therefore, did not create the obligation to prepare the plan but provided an evidence base for its subsequent development. Because this planning process began after the assessment period, it did not contribute to the effectiveness scores. Future assessments should examine whether the resulting plan translates the identified weaknesses into measurable targets, resource allocations, implementation responsibilities and monitoring arrangements. Bridging the output–outcome gap will ultimately depend not simply on implementing more activities but on using evidence to determine which actions are effective and how management should be adjusted over time.

## Conclusion

This study assessed the management effectiveness of the recently established Racha Islands MPA within a tourism-dependent coral-reef system in Thailand. The overall effectiveness score was 54.3%, indicating moderate performance, but performance varied substantially across the management cycle. Context and outputs were rated good, planning, process and outcomes were moderate and inputs were low. Legal designation, the general five-year management plan (2024–2028) and the delivery of many planned activities provided an important initial foundation. However, limited management inputs and partially functioning operational processes constrained the translation of these achievements into demonstrable ecological and socio-economic outcomes.

The Racha Islands MPA has progressed beyond legal designation but cannot yet demonstrate long-term conservation effectiveness. Insufficient and unstable funding, limited personnel and inadequate operational equipment constrained sustained enforcement, communication, monitoring and stakeholder engagement. Stakeholders participated in consultation during designation and in selected management activities, but participation had not extended to site-specific management planning or shared decision-making at the time of the assessment. Tourism-related behaviours and intensive vessel and recreational activities continued to present governance pressures, while threats were perceived as broadly unchanged, ecological trends remained inconsistent and evidence of socio-economic change was limited. Management outputs should therefore not be treated as equivalent to achieved conservation outcomes.

The next stage of MPA development should prioritize predictable financing, adequate staffing and equipment, development and implementation of the required site-specific management plan through meaningful stakeholder engagement, an operational and representative advisory mechanism, strengthened tourism communication and compliance arrangements, and a feasible set of ecological, socio-economic and governance indicators linked to management objectives. These measures would enable management authorities to evaluate results, learn from implementation and adjust management strategies over time. Post-assessment steps to convene the advisory committee and begin preparing the legally required site-specific plan using the assessment findings represent encouraging institutional responses, but these developments occurred after the scoring period and their effectiveness remains to be evaluated. More broadly, the study demonstrates that early-stage MPA development is an uneven progression from legal establishment to operational implementation and, ultimately, to measurable outcomes. This lesson is particularly relevant to tourism-dependent coastal regions where intensive resource use places continuing demands on newly established conservation institutions.

## Supplementary information

ppendix A_Table_A1_A2

Appendix B

Appendix C

## Data Availability

The participant-level data generated and analysed during the current study are not publicly available due to confidentiality and ethical restrictions. De-identified data may be available from the corresponding author upon reasonable request, subject to applicable ethical requirements.
