# Supplementary material for: Bridging the Gap Between Legal Designation and Management Outcomes: Evidence from a Newly Established Marine Protected Area in Thailand
Source: Environ Manage. 2026 Sep 24;76(10):330. doi: 10.1007/s00267-026-02622-x (PMC13612671; doi:10.1007/s00267-026-02622-x)
Supplement: Supplementary file 1 [file 267_2026_2622_MOESM1_ESM.docx]

**Appendix A**

**Table A1.** Indicator-specific scoring criteria used in the management effectiveness assessment

Each indicator was scored from 0 to 3 using the operational criteria below. The criteria were translated and standardised from the assessment protocol used in the Thai technical report. Final scores were assigned after triangulating documentary evidence, stakeholder questionnaires and interviews, field observations, and available ecological information.

| **Code** | **Indicator** | **0** | **1** | **2** | **3** |
| --- | --- | --- | --- | --- | --- |
| **Context (5 indicators)** | | | | | |
| Co1 | Legal establishment of the marine protected area | No formal designation or legal recognition. | Legal designation is being prepared. | Legally designated, but significant establishment-related conflict remains. | Legally designated and accepted without significant establishment-related conflict. |
| Co2 | Clarity of protected area boundaries | Boundaries are not formally defined. | Boundaries are legally mapped, but no physical markers are present. | Boundaries are legally mapped and partly marked in the field. | Boundaries are legally mapped, fully marked, and clearly communicated. |
| Co3 | Stakeholder awareness of the ecological value and status of marine and coastal resources | Stakeholders show little or no understanding of resource values or condition. | Understanding is limited and confined to a small proportion of stakeholders. | Moderate understanding is evident, but knowledge is uneven among groups. | Key stakeholder groups demonstrate strong understanding of resource values and condition. |
| Co4 | Stakeholder awareness and management of threats to marine and coastal resources | Major threats are not recognised. | Threats are recognised, but no management response is evident. | Threats are recognised and responses exist, but pressures have not clearly declined. | Threats are recognised and effectively addressed, with clear evidence of reduction. |
| Co5 | Identification and engagement of stakeholder groups involved in management | Relevant stakeholder groups have not been identified. | Stakeholders are recognised, but no consultation or joint work has occurred. | Stakeholders are identified and occasionally consulted or involved in joint activities. | Stakeholders are systematically identified and regularly involved in management. |
| **Planning (8 indicators)** | | | | | |
| Pl1 | Adequacy of management policies and strategies | No relevant management policy or strategy exists. | Broad policy exists but cannot guide operational planning. | Policy provides guidance for most management planning needs. | Clear and comprehensive policy provides a consistent basis for management planning. |
| Pl2 | Appropriateness of protected area design, size, boundaries, and ecological connectivity | Design is poorly aligned with ecological processes and area requirements. | Some ecological considerations are included, but important design limitations remain. | Design broadly reflects ecological connectivity and appropriate spatial scale. | Design is strongly justified by ecological connectivity, conservation objectives, and appropriate scale. |
| Pl3 | Clarity and measurability of management objectives | Objectives are absent, unclear, or impracticable. | Objectives are stated but broad and not measurable. | Objectives are clear and actionable, but only partly measurable. | Objectives are clear, actionable, and linked to measurable indicators and targets. |
| Pl4 | Existence of a systematic management planning process | No structured management-planning process exists. | A plan exists, but project-cycle integration and multi-year operational continuity are limited. | Planning covers most stages of the management cycle and includes some multi-year continuity. | A complete, long-term, adaptive planning cycle links analysis, resources, implementation, monitoring, and review. |
| Pl5 | Integration and coordination with relevant agencies | No coordination or alignment with relevant agencies. | Some alignment exists, but substantial duplication or coordination problems remain. | Most agency plans are aligned and duplication is limited. | Strong inter-agency alignment and coordination occur without important duplication or conflict. |
| Pl6 | Planning for threat reduction, restoration, and capacity enhancement | No plan addresses threat reduction, restoration, or resilience/capacity enhancement. | The plan addresses only one of these areas. | The plan addresses multiple areas, but coverage or feasibility is incomplete. | Threat reduction, restoration, and capacity enhancement are comprehensively planned and feasible. |
| Pl7 | Existence and completeness of action plans | No action plan exists. | An action plan exists, but responsibilities, steps, resources, or timing are unclear or incomplete. | Most action-plan elements are specified, with minor gaps. | The action plan clearly specifies activities, responsibilities, resources, timing, and expected outputs. |
| Pl8 | Stakeholder participation in management planning | Stakeholders do not participate in management planning. | Stakeholders participate only as information providers. | Stakeholders provide information and advice during planning. | Stakeholders provide information and advice and hold formal planning or decision-making roles. |
| **Inputs (7 indicators)** | | | | | |
| Ip1 | Adequacy of the management budget | Budget is insufficient for both administration and planned management activities. | Budget covers basic administration but not planned activities. | Budget covers administration and some planned activities. | Budget is sufficient for administration and all planned activities. |
| Ip2 | Stability and sustainability of financial support | No reliable or predictable funding source exists. | Funding depends on one short-term or annual source. | One main source provides reasonably stable multi-year support. | Stable long-term public funding is complemented by additional funding sources. |
| Ip3 | Adequacy of personnel for management activities | No personnel are available to implement the management plan. | Personnel are sufficient for only some planned activities. | Personnel are sufficient for most planned activities. | Personnel are sufficient for all planned activities and routine management functions. |
| Ip4 | Staff skills and technical capacity | Staff lack the knowledge and skills required for planned activities. | Staff capacity is sufficient for only some activities. | Staff capacity is sufficient for most activities, with identifiable gaps. | Staff possess the knowledge and skills required for all major activities. |
| Ip5 | Availability of baseline physical, biological, economic, and social information | No relevant baseline information or research is available. | Available information is very limited and inadequate for planning or decisions. | Moderate information is available and supports some planning and decisions, but gaps remain. | Comprehensive physical, biological, social, and economic information supports planning, decisions, and future assessment. |
| Ip6 | Support from the private sector, NGOs, stakeholders, and local communities | No support is provided by private-sector organisations, stakeholders, or local communities. | Support is provided occasionally (approximately 1-2 instances). | Support is provided repeatedly (approximately 3-4 instances), but remains uneven among groups. | Government, private-sector, stakeholder, and community support is sustained every year. |
| Ip7 | Adequacy and operational condition of management equipment and facilities | Essential equipment and facilities are insufficient or non-operational. | Equipment is broadly sufficient, but much is in poor condition. | Equipment is broadly sufficient and mostly operational. | Equipment and facilities are sufficient, well maintained, and consistently operational. |
| **Process (11 indicators)** | | | | | |
| Pr1 | Communication and clarification of management responsibilities | No management briefing or clarification occurs. | Briefing occurs only within the managing agency. | Internal and external briefings occur, but irregularly. | Responsibilities and procedures are regularly communicated to staff and relevant external actors. |
| Pr2 | Stakeholder participation in management activities | Stakeholders do not participate in ongoing management activities. | Only a small number of stakeholder groups participate. | Several groups participate, but involvement is uneven or only partly effective. | Multiple stakeholder groups participate regularly and effectively. |
| Pr3 | Good governance and transparency in management | No transparent oversight or coordination mechanism is evident. | An oversight arrangement exists but has limited acceptance or credibility. | Governance arrangements are functioning, but authority, representation, or transparency remains limited. | Inclusive oversight has clear authority, transparency, accountability, and decision-making influence. |
| Pr4 | Monitoring and evaluation of management implementation | No monitoring or evaluation of implementation progress occurs. | Monitoring is incomplete and no formal progress evaluation is undertaken. | Most activities are monitored, but progress evaluation is irregular or occurs only about once a year. | Implementation is comprehensively monitored and progress is evaluated at appropriate intervals (approximately every 3-6 months). |
| Pr5 | Staff training and capacity-building activities | No staff capacity-building is provided. | No training plan exists; training is only ad hoc or needs-based. | A training plan exists but is not fully implemented. | A training plan is implemented as scheduled and addresses management needs. |
| Pr6 | Maintenance of management equipment and facilities | No maintenance plan or routine maintenance occurs. | Maintenance is mainly reactive and undertaken only when necessary. | Planned maintenance is completed for most equipment. | Preventive maintenance is planned and implemented consistently. |
| Pr7 | Adequacy and enforcement of laws and regulations | Laws or regulations are inadequate for management needs. | Legal provisions exist, but enforcement is ineffective because of capacity or procedural constraints. | Enforcement is generally effective, although some constraints remain. | Laws and regulations are adequate and consistently enforced effectively. |
| Pr8 | Conflict-resolution mechanisms | No conflict-resolution process exists. | Conflicts are addressed only through informal negotiation. | A formal multi-party mechanism exists, but its operation is not yet effective. | A recognised multi-party mechanism resolves conflicts effectively. |
| Pr9 | Use of research and monitoring information in management decisions | No relevant research or monitoring information is available. | Information exists but is insufficient for management decisions. | Research or monitoring informs some management decisions. | Research and monitoring routinely inform most management decisions. |
| Pr10 | Public communication, outreach, and awareness-building activities | No outreach or public information is provided. | Limited outreach reaches only a small audience. | Several communication products or activities exist, but coverage remains incomplete. | Regular, accessible outreach reaches relevant stakeholder groups through multiple channels. |
| Pr11 | Dissemination of management performance and implementation results | Management results are not disseminated. | Results are shared only within the managing agency. | Results are shared with selected stakeholder groups. | Results are publicly and transparently disseminated through formal reporting and communication. |
| **Outputs (6 indicators)** | | | | | |
| Op1 | Implementation of planned management activities | Less than 25% of planned activities are implemented. | 25-<50% of planned activities are implemented. | 50-75% of planned activities are implemented. | More than 75% of planned activities are implemented. |
| Op2 | Timely implementation of management activities | Activities are substantially delayed. | Activities are slightly delayed. | Activities are completed within the planned timeframe. | Activities are completed ahead of schedule. |
| Op3 | Delivery of management outputs and services | No intended outputs or services are delivered. | Only a small proportion of intended outputs is delivered. | Most intended outputs are delivered, with some gaps. | All intended outputs and services are delivered. |
| Op4 | Stakeholder understanding of marine protected area management | Less than 25% of stakeholders demonstrate adequate understanding of MPA management. | 25-<50% demonstrate adequate understanding. | 50-75% demonstrate adequate understanding. | More than 75% demonstrate adequate understanding. |
| Op5 | Stakeholder compliance with rules and regulations | Non-compliance is widespread and routine. | Compliance is low or inconsistent across groups and activities. | Most stakeholders comply, but recurrent violations are still reported or observed. | Compliance is consistently high, with few or no reported or observed violations. |
| Op6 | Staff and agency understanding of the management plan | Less than 25% of staff and relevant agency respondents understand the management plan. | 25-<50% understand the management plan. | 50-75% understand the management plan. | More than 75% understand the management plan. |
| **Outcomes (6 indicators)** | | | | | |
| Oc1 | Achievement of management objectives | Management objectives have not been achieved. | Some progress toward objectives is evident, but achievement remains limited. | Most objectives are close to being achieved or have been substantially achieved. | All major objectives have been achieved and verified. |
| Oc2 | Reduction of threats and environmental pressures | Threats and pressures are increasing. | Threats remain broadly unchanged. | Threats show a downward tendency, but evidence is not yet clear or consistent. | Threats have clearly and demonstrably declined. |
| Oc3 | Environmental sustainability of resource use | Environmental condition is deteriorating. | Environmental condition is broadly unchanged. | Environmental condition shows improvement, but the trend is not yet clear or consistent. | Environmental condition has clearly and consistently improved. |
| Oc4 | Socio-economic sustainability outcomes | Socio-economic conditions associated with resource use are worsening. | Socio-economic conditions are broadly unchanged. | Socio-economic conditions show some improvement. | Socio-economic conditions have clearly and broadly improved. |
| Oc5 | Stakeholder satisfaction with management and enforcement | Less than 25% of stakeholders are satisfied with MPA management. | 25-<50% are satisfied. | 50-75% are satisfied. | More than 75% are satisfied. |
| Oc6 | Partnerships and collaboration among stakeholders and relevant agencies | No management collaboration or partnership is evident. | Some actors cooperate occasionally, without an identifiable group or mechanism. | Most relevant actors cooperate through identifiable groups or joint activities, but arrangements are not fully institutionalised. | Formal, representative, and sustained partnership arrangements jointly support management and oversight. |

*Note. MPA = marine protected area.*

**Table A2.** Indicator scores and supporting evidence for the Racha Islands Marine Protected Area

Evidence sources are summarised below. The basis-for-score column presents the principal evidence supporting each final score. Component totals and the overall index were calculated from all 43 indicators.

| **Code** | **Indicator** | **Evidence sources** | **Score** | **Basis for score** |
| --- | --- | --- | --- | --- |
| **Context: 11/15 (73.3%)** | | | | |
| Co1 | Legal establishment of the marine protected area | Legal notification; establishment records; stakeholder survey | 3 | The MPA was legally designated in 2023 following stakeholder consultation, and no significant establishment-related conflict was identified. |
| Co2 | Clarity of protected area boundaries | Legal boundary map; stakeholder questionnaire and interviews; field observations | 1 | The boundary is formally mapped, but physical boundary markers are absent or incomplete; 54.8% of surveyed users considered the boundary unclear, particularly around the coral-reef zone. |
| Co3 | Stakeholder awareness of the ecological value and status of marine and coastal resources | Stakeholder questionnaire and interviews | 3 | Respondents showed strong awareness of the MPA’s ecological and use values: 60% identified its habitat function and 48% identified its importance for marine tourism. |
| Co4 | Stakeholder awareness and management of threats to marine and coastal resources | Stakeholder questionnaire and interviews; management records | 2 | More than 90% of respondents recognised continuing threats. Patrols, mooring buoys, zoning, and other responses were in place, but threats had not clearly declined and implementation remained constrained by budget and staffing. |
| Co5 | Identification and engagement of stakeholder groups involved in management | Institutional interviews; consultation and activity records | 2 | Government, tourism, fishing, community, conservation, and academic groups were recognised and had been consulted or involved in some activities, but engagement was not regular or comprehensive. |
| **Planning: 14/24 (58.3%)** | | | | |
| Pl1 | Adequacy of management policies and strategies | Policy and legal documents; five-year management plan; institutional interviews | 3 | Policy and legal instruments, together with the five-year management plan (2024–2028), provided clear strategic direction covering administration, monitoring, protection, restoration, and stakeholder participation. |
| Pl2 | Appropriateness of protected area design, size, boundaries, and ecological connectivity | Legal map; ecological information; management plan; institutional interviews | 3 | The legally designated area and three management zones were designed with reference to coral reefs, coastal habitats, ecological connectivity, and differentiated use controls. |
| Pl3 | Clarity and measurability of management objectives | Management objectives and plan; institutional interviews | 1 | Management objectives were stated and broadly actionable, but quantitative targets and measurable outcome indicators were largely absent. |
| Pl4 | Existence of a systematic management planning process | Management plan; annual work plans and reports; institutional interviews | 1 | A general five-year management plan existed, but detailed activities and budgets were prepared mainly on an annual basis, limiting multi-year operational continuity. |
| Pl5 | Integration and coordination with relevant agencies | Agency plans and documents; cross-agency interviews | 3 | Management responsibilities were aligned with relevant agencies, including the fisheries and marine-transport authorities, and no major duplication or conflict was identified. |
| Pl6 | Planning for threat reduction, restoration, and capacity enhancement | Management plan; activity records; institutional interviews | 2 | Plans covered patrols, mooring-buoy installation and repair, waste removal, coral restoration, and participation, but implementation was incomplete because of budget and staffing constraints. |
| Pl7 | Existence and completeness of action plans | Action plans and annual work plans; institutional interviews | 1 | Annual action plans specified activities and timing, but operational steps and other implementation details were incomplete. |
| Pl8 | Stakeholder participation in management planning | Five-year management plan and annual implementation plan; planning and consultation records; stakeholder questionnaire and interviews | 0 | Stakeholders were consulted during designation but had no substantive role in preparing either the five-year management plan or the annual implementation plan for fiscal year 2025. Interview respondents were generally unaware of the annual implementation plan. |
| **Inputs: 7/21 (33.3%)** | | | | |
| Ip1 | Adequacy of the management budget | Budget information; institutional interviews | 0 | Funding was insufficient for routine administration and planned field activities, including staffing, vessel operation, equipment repair, and mooring-buoy maintenance. |
| Ip2 | Stability and sustainability of financial support | Funding and annual budget information; institutional interviews | 1 | Funding depended on annual government allocations, with no secure multi-year budget or diversified financing source. |
| Ip3 | Adequacy of personnel for management activities | Staffing information; institutional interviews | 1 | The centre had six staff members, which was insufficient for the range and continuity of island-based, vessel, patrol, technical, and administrative duties. |
| Ip4 | Staff skills and technical capacity | Staff interviews; training and activity records | 1 | Because the MPA and management centre were newly established, staff had only partial management capacity and important operational skills still needed development. |
| Ip5 | Availability of baseline physical, biological, economic, and social information | Baseline studies and monitoring reports; institutional interviews | 2 | Ecological, water-quality, oceanographic, and navigation information was available from partner agencies, but socio-economic baseline data were lacking. |
| Ip6 | Support from the private sector, NGOs, stakeholders, and local communities | Stakeholder questionnaire and interviews; activity records | 2 | About 70% of surveyed users reported providing some support, especially diving and tourism groups, but support was uneven and many fishers reported never contributing resources. |
| Ip7 | Adequacy and operational condition of management equipment and facilities | Equipment information; field observations; institutional interviews | 0 | Essential equipment and facilities were insufficient: of the Centre’s two patrol vessels, one required repair, while the other—the only operational vessel—experienced frequent breakdowns. The temporary island office was not fully operational. |
| **Process: 16/33 (48.5%)** | | | | |
| Pr1 | Communication and clarification of management responsibilities | Meeting records; institutional interviews | 1 | Operational briefings were conducted mainly within the management centre and had not consistently reached external stakeholders. |
| Pr2 | Stakeholder participation in management activities | Stakeholder questionnaire; activity records; interviews | 2 | Several groups participated in buoy work, underwater clean-ups, restoration, and related activities; 58% reported participating, but involvement remained uneven and not fully effective. |
| Pr3 | Good governance and transparency in management | Governance documents; stakeholder questionnaire and interviews | 1 | An advisory committee had been appointed in March 2025, and some consultation and reporting mechanisms existed, but the committee had not yet operated and inclusive oversight remained limited. |
| Pr4 | Monitoring and evaluation of management implementation | Progress and implementation reports; institutional interviews | 3 | Implementation progress was monitored and reported approximately every 4-6 months, enabling problems and constraints to be reviewed. |
| Pr5 | Staff training and capacity-building activities | Training records; institutional interviews | 1 | No dedicated MPA capacity-building plan existed; training was limited mainly to immediate needs such as lifesaving rather than core management functions. |
| Pr6 | Maintenance of management equipment and facilities | Maintenance information; field observations; institutional interviews | 1 | No preventive-maintenance plan was documented; repairs and maintenance were undertaken mainly when problems arose. |
| Pr7 | Adequacy and enforcement of laws and regulations | Legal instruments; enforcement information; institutional interviews | 1 | Legal authority was considered adequate, but staff uncertainty about procedures and enforcement steps reduced effective implementation. |
| Pr8 | Conflict-resolution mechanisms | Stakeholder questionnaire and interviews; committee information | 1 | An advisory committee had been appointed, but it was not yet operational; conflict resolution therefore remained largely informal and was not widely recognised as effective. |
| Pr9 | Use of research and monitoring information in management decisions | Research and monitoring reports; institutional interviews | 2 | The centre had no systematic internal research programme, but external coral-reef and water-quality monitoring informed some decisions, including coral restoration. |
| Pr10 | Public communication, outreach, and awareness-building activities | Communication materials; field observations; stakeholder questionnaire and interviews | 1 | Signs and limited outreach were present, but coverage and communication channels were narrow; about 63% of respondents considered communication ineffective or insufficient. |
| Pr11 | Dissemination of management performance and implementation results | Progress reports; meeting records; institutional interviews | 2 | Performance was reported to selected committees and meeting participants, while wider public reporting and social-media dissemination were still being developed. |
| **Outputs: 12/18 (66.7%)** | | | | |
| Op1 | Implementation of planned management activities | Implementation and activity reports; institutional interviews | 3 | More than 75% of planned activity categories had been implemented, although some activities depended on support from partner agencies and stakeholders. |
| Op2 | Timely implementation of management activities | Activity schedules and reports; institutional interviews | 2 | Activities were generally completed within the planned timeframe, although some were incomplete in scope or required external support. |
| Op3 | Delivery of management outputs and services | Output and activity reports; field observations; institutional interviews | 2 | Most intended outputs were delivered, but some outputs remained incomplete because of budget constraints. |
| Op4 | Stakeholder understanding of marine protected area management | Stakeholder questionnaire | 2 | Approximately 54.3% of surveyed users reported substantial or strong understanding of MPA management, while sizeable knowledge gaps remained. |
| Op5 | Stakeholder compliance with rules and regulations | Stakeholder questionnaire and interviews | 2 | Self-reported compliance was high (83.7%), but respondents still reported recurring violations, including anchoring on coral reefs and fish feeding; the triangulated score therefore remained. |
| Op6 | Staff and agency understanding of the management plan | Institutional interviews | 1 | Fewer than half of relevant institutional respondents demonstrated understanding of the management plan, and several had seen the plan without understanding its details. |
| **Outcomes: 10/18 (55.6%)** | | | | |
| Oc1 | Achievement of management objectives | Implementation reports; institutional interviews | 1 | Some progress was evident, including indications of reduced violations, but the absence of quantitative indicators and targets prevented verification of broad objective achievement. |
| Oc2 | Reduction of threats and environmental pressures | Management records; stakeholder questionnaire and interviews | 1 | Management actions appeared to reduce some harmful behaviours, but most respondents perceived the overall level of threats as broadly unchanged and no quantitative threat indicators were available. |
| Oc3 | Environmental sustainability of resource use | Ecological monitoring; stakeholder questionnaire and interviews | 2 | Ecological monitoring indicated stable or improving conditions at several sites, but some coral areas deteriorated and trends were not yet clear or consistent across the MPA. |
| Oc4 | Socio-economic sustainability outcomes | Stakeholder questionnaire and interviews | 1 | Most respondents reported little change in income or socio-economic conditions, and no pre-designation baseline or formal socio-economic indicators were available. |
| Oc5 | Stakeholder satisfaction with management and enforcement | Stakeholder questionnaire | 3 | More than 80% of surveyed users were satisfied with MPA management and enforcement. |
| Oc6 | Partnerships and collaboration among stakeholders and relevant agencies | Stakeholder and institutional interviews; joint-activity records | 2 | Local conservation groups and stakeholders collaborated in activities, but cooperation remained informal and was not yet supported by a fully operational joint management mechanism. |
|  | **Overall management effectiveness** |  | **70/129** | **Overall management effectiveness = 54.3% (moderate performance).** |

*Note. MPA = marine protected area.*
