# Supplementary material for: Bridging the Gap Between Legal Designation and Management Outcomes: Evidence from a Newly Established Marine Protected Area in Thailand
Source: Environ Manage. 2026 Sep 24;76(10):330. doi: 10.1007/s00267-026-02622-x (PMC13612671; doi:10.1007/s00267-026-02622-x)
Supplement: Supplementary file 2 [file 267_2026_2622_MOESM2_ESM.docx]

**Appendix B**. Summary of the questionnaire and semi-structured interview guide used with local resource users and tourism-related stakeholders

Section 1. Respondent characteristics

This section collected general demographic and socio-economic information, including gender, age, education level, place of residence, type of resource use, and duration of involvement within the marine protected area. During semi-structured interviews, the questions in Sections 2-7 were used as prompts, and respondents were invited to explain the reasons and experiences underlying their questionnaire responses.

Section 2. Context

Are you aware that this area has been designated as a marine protected area? (Co1)

How clear are the boundaries of the marine protected area? (Co2)

Are you aware of the ecological importance and conservation value of the area? (Co3)

Are you aware of major threats affecting marine and coastal resources in the area? (Co4)

Section 3. Planning

To what extent have you participated in management planning for the marine protected area? (Pl8)

Section 4. Inputs

Have you contributed resources or support to marine protected area management activities? (Ip6)

Section 5. Process

To what extent have you participated in management activities? (Pr2)

How transparent and fair is the management of the marine protected area? (Pr3)

Has conflict resolution related to resource use improved after the establishment of the marine protected area? (Pr8)

How effective is communication and public outreach regarding marine protected area management? (Pr10)

Section 6. Outputs

How well do you understand the objectives, regulations, and management plans of the marine protected area? (Op4)

To what extent do you comply with the rules and regulations of the marine protected area? (Op5)

Section 7. Outcomes

Have environmental threats and resource-related problems changed since the establishment of the marine protected area? (Oc2)

How have marine biodiversity, habitats, and fisheries resources changed following the establishment of the marine protected area? (Oc3)

How has the marine protected area affected your income or livelihood? (Oc4)

How satisfied are you with the MPA management and enforcement? (Oc5)

To what extent has collaboration among stakeholders improved within the marine protected area? (Oc6)
