# Supplementary material for: Bridging the Gap Between Legal Designation and Management Outcomes: Evidence from a Newly Established Marine Protected Area in Thailand
Source: Environ Manage. 2026 Sep 24;76(10):330. doi: 10.1007/s00267-026-02622-x (PMC13612671; doi:10.1007/s00267-026-02622-x)
Supplement: Supplementary file 3 [file 267_2026_2622_MOESM3_ESM.docx]

**Appendix C. Summary of the semi-structured in-depth interview guide used with institutional respondents**

The interview guide was used to collect in-depth qualitative evidence on institutional arrangements, governance processes, implementation constraints, and management outcomes. The themes informed the assessment and interpretation of relevant indicators across the IUCN-WCPA management effectiveness framework.

**Section 1. Respondent background**

This section collected information on respondents’ institutional affiliation, professional role, area of responsibility, and duration of involvement with the marine protected area.

**Section 2. Marine protected area management**

| Code | Interview theme | Example focus |
| --- | --- | --- |
| Co4 | Ecological conditions and threats | Perceptions of ecosystem status and major environmental pressures |
| Pl5 | Inter-agency coordination \| | Alignment or conflicts between management plans and agency responsibilities |
| Pl8 | Participation in planning | Stakeholder involvement in management planning and decision-making |
| Ip6 | Resource support | Contributions of financial, technical, or institutional support |
| Pr1 | Management communication | Clarity of management roles and responsibilities, and participation in meetings and coordination processes |
| Pr10 | Public communication and outreach | Effectiveness of communication and information dissemination |
| Oc6 | Collaboration and partnerships | Cooperation among agencies, stakeholders, and local communities |

**Section 3. Recommendations for management improvement**

Overall management effectiveness: Perceptions of governance effectiveness and sustainability outcomes

Governance challenges: Institutional, operational, and coordination constraints

Recommendations: Suggestions for improving long-term management and sustainability
